# Caring for a child with cancer during COVID-19 pandemic: an assessment of the parents’ perception and stress level

**DOI:** 10.3389/fpubh.2024.1223362

**Published:** 2024-04-08

**Authors:** Muhamad Aizat Nawi, Sie Chong Doris Lau, Shi Tying Chin, Kok Hoi Teh, Lee Sue Betty Ho, Hamidah Alias

**Affiliations:** ^1^Department of Paediatrics, Faculty of Medicine, Universiti Kebangsaan Malaysia Medical Centre, Kuala Lumpur, Malaysia; ^2^Department of Paediatrics, Sarawak General Hospital, Kuching, Malaysia; ^3^Department of Paediatrics, Hospital Tunku Azizah, Kuala Lumpur, Malaysia

**Keywords:** COVID-19, children, cancer, perception, paediatric oncology

## Abstract

**Background:**

The emergence of COVID-19 pandemic has led to heightened fear and uncertainty among parents of children with cancer. This study was conducted to evaluate the parental perceptions toward effects of COVID-19 infection to children with cancer, determine their stress level and factors contributing to high stress level during the pandemic.

**Methods:**

This cross-sectional study was conducted in three paediatric oncology centres in Malaysia from September 2020 until December 2022. A total of 167 parents were recruited. Parents completed a set of questionnaires to assess their perception on effect of COVID-19 infection to children with cancer and COVID Stress Scale (CSS) to assess the parents’ stress level.

**Results:**

Patients’ mean age at study entry was 8.75 years (SD 4.38). Ninety-one (54.5%) patients were still on active treatment. More than 80% of the parents obtained information regarding COVID-19 infection from mass media and social networking. Fear of their children contracting COVID-19 infection was high especially among patients who were still on treatment. Forty-nine (29.3%) parents were significantly affected by the pandemic leading to loss of job or monthly income. Twenty-nine (17.4%) patients required treatment modification during the pandemic. The median total score for CSS was 78.0 (IQR 25^th^ 64.0; 75^th^ 95.0). Ninety-one (54.5%) respondents were very/extremely stressed based on the CSS scores. Components with high scores were xenophobia (median score 18.0; IQR 25^th^ 13.0, 75^th^ 22.0), fear of danger (median score 17.0; IQR 25^th^ 14.0, 75^th^ 20.0) and contamination fears (median score 16.0; IQR 25^th^ 12.0, 75^th^ 19.0). Lower household income was associated with higher stress level (*p* = 0.006).

**Conclusion:**

Our study demonstrated high awareness regarding risk of COVID-19 infection among parents of oncology children. Half of the parents had high stress level, with low household income identified as a factor associated with high stress level.

## 1 Introduction

COVID-19 was declared as a global pandemic by the World Health Organization (WHO) on 11 March 2020 (1). Up to March 2023, a total of 750 million cases have been confirmed worldwide and 6.8 million deaths reported, leading to a significant impact to the global healthcare and medical system (2). Nearly 90% of children with COVID-19 presented with mild or moderate symptoms, with no increased risk of morbidity nor mortality among children with cancer compared to the general population (3–5). However, diversion of healthcare system worldwide to cope with the pandemic has affected care for paediatric oncology patients, such as delayed presentation to hospital, interruptions in treatment protocol, shortage of blood supply, and suspensions of clinical trials for children with cancer (6–8).

In Malaysia, paediatric oncology services are only offered in dedicated centres. A great number of patients have to travel across states to the nearest oncology centre available for their treatment. During COVID-19 pandemic, patients who were newly diagnosed and receiving intensive phase of chemotherapy were greatly affected as their chemotherapy intervals were more frequent and experts were unsure whether adjustment to treatment protocols will affect the disease outcomes. Although planned treatment for children with cancer should continue to be delivered in a timely manner, a few modifications were necessary during the pandemic, considering the patients’ safety and service constraints.

COVID-19 pandemic has led to increased psychosocial stress in general population; parents of children with cancer have been reported to experience additional burden (9, 10). Various concerns were raised by parents, namely fear of COVID-19 infection compromising their child’s immune system further following intensive chemotherapy, as well as concerns regarding the risk of relapse if chemotherapy was delayed or treatment protocol was modified (11, 12). The lack of specific information on the potential threat of COVID-19 infection to children and adolescents with cancer may potentiate the stress and anxiety level among the parents. Thus, it is important to identify the parents’ anxiety and stress level to ensure that continuous open communication was made to address their concerns. In this study, we evaluated the parents’ perception towards effects of COVID-19 infection in children with cancer, determined their stress level and factors contributing to high stress level during the pandemic.

## 2 Methods

This study was carried out over a 28 months period (from 1^st^ September 2020 until 31^st^ December 2022) at three paediatric oncology centres in Malaysia – Universiti Kebangsaan Malaysia Medical Centre (UKMMC), Sarawak General Hospital (SGH) and Hospital Tunku Azizah (HTA). Parents (either mother or father) of children or adolescent (<18 years old) with cancer who were receiving treatment or follow up at one of the aforementioned centres were included in the study. Patients who were on palliative care and foreigners were excluded. Convenience sampling was employed and eligible parents were approached, either in the paediatric oncology ward or daycare when their child was admitted for chemotherapy, or in the oncology clinic for patients who have completed treatment. Written informed consent was obtained prior to recruitment. Ethics approval was obtained from the Research Ethics Committee, The National University of Malaysia (JEP-2021-028) and The Ministry of Health Medical Research Ethics Committee (MREC) of Malaysia (NMRR-20-3028-56868) prior to the study.

### 2.1 Questionnaire

Parents (either father or mother) were asked to complete a set of printed questionnaires. The questionnaires consisted of (i) demographic data, (ii) perception towards effects of COVID-19 infection/pandemic to their child, (iii) experience and difficulty faced while taking care of their child during pandemic (iv) treatment modification and their acceptance, and (v) COVID Stress Scale (CSS). Patients’ clinical data and treatment protocol were extracted from their medical records. The questionnaire pertaining to parental perception towards effects of COVID-19 infection to their child with cancer was adapted with permission from Casanova et al. (13). The original questionnaire was used to assess young cancer patients’ perception of COVID-19 infection risk (13). After expert group discussion among the paediatric oncologists from the three centres involved, modifications were made to this questionnaire to suit the sample population of this study (Supplementary Data Sheet). Forward translation to Bahasa Malaysia was performed and reliability of the translated questionnaire was performed prior to its use. Meanwhile, the parents’ stress level was assessed using COVID-19 Stress Scale (CSS) (Malay version) with permission from the original author, Taylor et al. (14, 15). The CSS had 6 major components, i.e., fear of danger, socioeconomic consequences, xenophobia, contamination fear, traumatic stress and compulsive checking. Each component has 6 questions. Likert scale (0 to 4; 0 indicating “Never/Not at all” while 4 indicating “Almost always/Extremely”) was used to score each of the questions. Total score of CSS is 144. The total scores were further categorised as follows: slightly stressed (≤36), moderately stressed (37–76), very stressed (77–108), extremely stressed (≥109).

### 2.2 Statistical analysis

Data was entered and analysed using SPSS version 26.0 (IBM Corp, Armonk, NY, United States). Frequency and percentage were calculated for categorical variables; mean and standard deviation were calculated for continuous variables. Chi-square (*X*^2^) test was used to test association between the categorical data. Spearman’s rho correlation was used to find the correlation between continuous data. Significance level was set at a *p* value of <0.05.

## 3 Results

A total of 167 parents completed the questionnaire, of which the majority were mothers (*n* = 135, 80.8%). Patients’ mean age at study entry was 8.75 years (SD 4.38) (Table 1). Ninety-one (54.5%) patients were still on active treatment; fifty-nine (64.8%) patients were on intensive phase chemotherapy. Almost two-third (*n* = 106; 63.5%) of the patients were from the lower socio-economic group. Thirty-eight (22.7%) patients had contact with another COVID-19 patient. Majority of the parents (>80%) obtained information regarding COVID-19 infection from mass media and social networking (e.g., Facebook, Instagram, Twitter) (Table 2). However, most of the information obtained was pertaining to the general population; seventy-five (44.9%) parents knew the effects of COVID-19 infection on children diagnosed with cancer.

**Table 1:** Demographic and clinical characteristics of study population.

Characteristic	Results (*N* = 167)
Patients’ age at study entry in years, mean (SD)	8.75 (4.38)
Gender, *n* (%)	
Male	100 (59.9)
Female	67 (40.1)
Ethnicity, *n* (%)	
Malay	130 (77.8)
Chinese	10 (6.0)
Indian	5 (3.0)
Others	22 (13.2)
Patients’ education level	
Not yet schooling	48 (28.7)
Pre-school/Kindergarten	19 (11.4)
Primary school	67 (40.1)
Secondary school	30 (18.0)
Others	3 (1.8)
Mothers’ age in years, mean (SD)	37.59 (6.73)
Fathers’ age in years, mean (SD)	39.67 (7.22)
Mothers’ education level, *n* (%)	
Primary school	9 (5.4)
Secondary school	77 (46.1)
Tertiary (University/college)	78 (46.7)
Others	3 (1.8)
Fathers’ education level, *n* (%)	
Primary school	8 (4.8)
Secondary school	92 (55.1)
Tertiary (University/college)	62 (37.1)
Others	5 (3.0)
Combined monthly household income, *n* (%)	
≤ RM4849 (B40)	106 (63.5)
RM4850-RM10959 (M40)	52 (31.1)
≥ RM10960 (T20)	9 (5.4)
Diagnosis, *n* (%)	
Leukaemia	96 (57.5)
Lymphoma	14 (8.4)
Brain tumour	12 (7.2)
Bone tumour	10 (6.0)
Neuroblastoma	9 (5.4)
Nephroblastoma	6 (3.6)
Retinoblastoma	5 (3.0)
Others	15 (9.0)
Patients’ treatment phase, *n* (%)	
On treatment	91 (54.5)
-Intensive phase	59 (64.8)
-Maintenance phase	32 (35.2)
Completed (off) treatment	76 (45.5)

*N*, numbers; SD, standard deviation; RM, ringgit Malaysia.

**Table 2:** Parental knowledge and perception on risk of COVID-19 infection to their child.

Items	Total (*N* = 167)	On treatment (*n* = 91)	Off treatment (*n* = 76)
Source of information on COVID-19 infection^§^			
–Broadcast media –Social networks –Press conferences/government statements –Family members/friends –Health professionals	145 (86.8%) 137 (82.0%) 136 (81.4%) 80 (47.9%) 52 (31.1%)	80 (87.8%) 74 (81.3%) 75 (82.4%) 48 (52.7%) 29 (31.9%)	65 (85.5%) 63 (82.9%) 61 (80.3%) 32 (42.1%) 23 (30.3%)
Knowledge on COVID-19 infection^§^			
–General knowledge –Specific complications to paediatric patients –Specific complications to cancer patients –Steps to reduce risk of child getting infected	142 (85.0%) 87 (52.1%) 75 (44.9%) 104 (62.3%)	79 (86.8%) 51 (56.0%) 42 (46.1%) 55 (60.4%)	63 (72.9%) 36 (47.4%) 33 (43.4%) 49 (64.5%)
Feels that COVID-19 pandemic is dangerous to child diagnosed with cancer			
–Dangerous –Moderately/slightly dangerous –Not at all	144 (86.2%) 20 (12.0%) 3 (1.8%)	76 (83.5%) 14 (15.4%) 1 (1.1%)	68 (89.5%) 6 (7.9%) 2 (2.6%)
Fear that child will catch COVID-19 infection			
–Very afraid –Moderately afraid –A little afraid –Not at all afraid	138 (82.6%) 16 (9.6%) 11 (6.6%) 2 (1.2%)	70 (76.9%) 10 (11.0%) 10 (11.0%) 1 (1.1%)	68 (89.5%) 6 (7.9%) 1 (1.3%) 1 (1.3%)
Fear that child may get severe complication if get COVID-19 infection			
–Very afraid –Moderately afraid –A little afraid –Not at all afraid	148 (88.6%) 12 (7.2%) 6 (3.6%) 1 (0.6%)	76 (83.5%) 10 (11.0%) 4 (4.4%) 1 (1.1%)	72 (94.8%) 2 (2.6%) 2 (2.6%) 0 (0.0%)

N, numbers. ^§^Parents allowed to answer more than one option.

### 3.1 Parental perception and impact of COVID-19 infection

Fear of their children contracting COVID-19 infection was high especially among patients who were still on treatment. More than 90% of parents agreed COVID-19 infection was dangerous to their child and worry if they experience serious complications from it, regardless whether their children were still on treatment or had already completed treatment. Parents also observed protective measures (more frequent hand washing and wearing facemask) taken by the medical personnel to reduce the spread of COVID-19 infection, especially among patients who were still on treatment.

Forty-nine (29.3%) parents were greatly affected by the pandemic leading to loss of job or monthly income, necessitating the help from relatives or hospital’s Medical Social Work Service to support their child’s treatment costs (Table 3). Forty-two (25.1%) parents faced difficulty bringing their child to hospital for treatment or follow up due to movement control order (MCO) implemented by the government. Most of the patients (69.5%) did not require any treatment modification during the pandemic. Among the patients who were still on treatment, seven patients required postponement of their chemotherapy date while one had his chemotherapy dosage reduced. Meanwhile, surveillance (imaging and blood monitoring) for fourteen patients who had completed treatment were delayed during the MCO.

**Table 3:** Impact of COVID-19 pandemic to the child’s care.

Items	Total (*N* = 167)	On treatment (*n* = 91)	Off treatment (*n* = 76)
Changes to daily habits to reduce risk of COVID-19 infection			
–Yes –No	140 (83.8%) 27 (16.2%)	75 (82.4%) 16 (17.6%)	65 (85.5%) 11 (14.5%)
Observed changes to medical personnel’s practices^§^			
–More frequent hand washing –Wearing mask all the time –Social distancing	109 (65.3%) 122 (73.1%) 112 (67.1%)	67 (73.6%) 73 (80.2%) 53 (58.2%)	42 (55.3%) 49 (64.5%) 59 (77.6%)
Difficult to bring child to hospital			
–Yes –No	42 (25.1%) 125 (74.9%)	28 (30.8%) 63 (69.2%)	14 (18.4%) 62 (81.6%)
Difficulties encountered^§^			
–Public transport services not available –Feeling unsafe to use public transport services –Private transport service (taxi/GRAB) too expensive –Police road block –Others	4 (9.5%) 10 (23.8%) 13 (30.9%) 18 (42.8%) 4 (9.5%)	3 (10.7%) 7 (25.0%) 7 (25.0%) 12 (42.9%) 3 (10.7%)	1 (7.1%) 3 (21.4%) 6 (42.9%) 6 (42.9%) 1 (7.1%)
Modification to child’s treatment plan/follow-up			
–Yes –No –Not aware	29 (17.4%) 116 (69.5%) 22 (13.2%)	15 (16.5%) 54 (59.3%) 22 (24.2%)	14 (18.4%) 62 (81.6%) 0 (0.0%)
Impact on parents’ financial status			
–Major impact –Slight impact –No impact	49 (29.3%) 95 (56.9%) 23 (13.8%)	29 (31.9%) 51 (56.0%) 11 (12.1%)	20 (26.3%) 44 (57.9%) 12 (15.8%)
Types of major impact^§*^			
–Lost job and income –Use of retirement money –Required assistance from hospital’s Social Work Services	19 (38.8%) 24 (49.0%) 12 (24.5%)	14 (48.3%) 13 (44.8%) 11 (37.9%)	5 (25.0%) 11 (55.0%) 1 (5.0%)

N, numbers. ^§^Parents allowed to answer more than one option (^*^two respondents from the off-treatment group did not answer this).

### 3.2 Parental stress level – COVID stress scale

**Figure 1:**
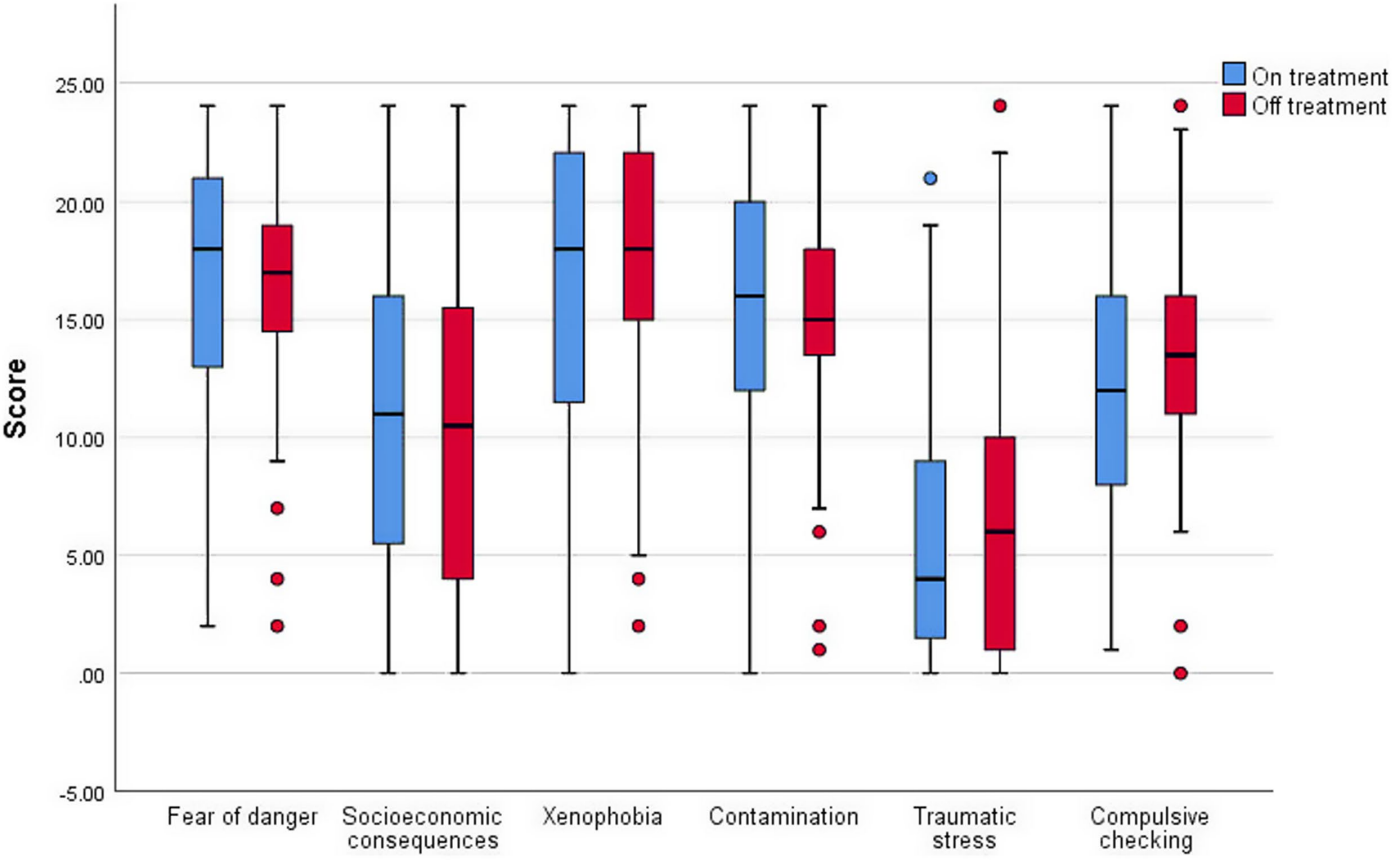
Scores for components of COVID-19 stress scale for on- and off-treatment patients.

The mean and median total score for CSS were 78.01 (SD 25.73) and 78.0 (IQR 25^th^ 64.0; 75^th^ 95.0) respectively (Supplementary Table S1). Seventy-one (42.5%) parents were in the ‘very stressed’ category, followed by 65 (38.9%) parents in the ‘moderately stressed’ category. Twenty (12.0%) parents were extremely stressed while the remaining eleven (6.6%) parents were slightly stressed. Components with high scores were xenophobia (median score 18.0; IQR 25^th^ 13.0, 75^th^ 22.0), danger (median score 17.0; IQR 25^th^ 14.0, 75^th^ 20.0) and contamination fears (median score 16.0; IQR 25^th^ 12.0, 75^th^ 19.0) (Figure 1). Meanwhile, the score for traumatic stress symptoms was lowest (median score 5.0; IQR 25^th^ 1.0, 75^th^ 10.0). Mean scores for each item in different components were tabulated in Supplementary Table S2. Further analysis revealed that lower household income was significantly associated with higher CSS score (Table 4).

**Table 4:** Correlation between CSS score and demographic parameters.

Factors	Total Score CSS (Spearman’s rho correlation)	
	*r*	*p* value
Patients’ age	0.006	0.852
Fathers’ education	−0.048	0.538
Mothers’ education	0.025	0.755
Total household income	−0.213	0.006^*^

CSS, COVID stress scale. ^*^Significant *p* < 0.05.

## 4 Discussion

The COVID-19 pandemic emerged in Wuhan, China, in late 2019, leading to widespread panic and global healthcare crisis. International health organisations such as World Health Organisation (WHO) and Centres for Disease Control and Prevention (CDC) releases periodic updates during the height of the pandemic to increase public awareness regarding COVID-19 infections. Parents, especially those with children suffering from chronic illnesses, would probably be highly aware of the consequences of severe COVID-19 infection and actively seeking ways to protect their children from the infection. A study by Sabra et al. among parents of general population in Egypt, found that most of participants (86.8%) perceived COVID-19 as a serious disease and were concerned about themselves or a family member being infected with COVID-19 (16). The current study also revealed similar findings; almost all the parents (98%) perceived COVID-19 to be a danger to their child and more than 90% were worried their child will get severe complications from COVID-19 infection. A change in daily habits were observed among a majority (83.8%) of the parents to reduce the risk of getting COVID-19 infection, in line with the guidelines released by CDC and WHO to reduce transmission of COVID-19 virus (i.e., masking, physical distancing, hand hygiene) (17).

Diversion of healthcare services to handle COVID-19 patients were implemented worldwide during the pandemic. This, together with movement restriction, has necessitates adjustment of treatment protocols among paediatric oncology patients in some countries (18). Due to limited data available, clinicians were in dilemma whether to continue anticancer treatment in patients with positive COVID-19 test result (18). Risk of cancer progressing or relapse due to interruption of chemotherapy has to be weighed against the risk of severe COVID-19 disease with potentially fatal outcome. Several studies recommend a multidisciplinary decision approach on treatment postponement, modification, or continuation in these situations, taking into consideration the clinical course of COVID-19 infection as well as patients’ existing comorbidities (19–22). Although early data showed continuation of chemotherapy in paediatric cancer patients with COVID-19 infection seems possible, more data is needed before solid recommendations can be made (19, 23). In current study, only a few patients required adjustment in their treatment and follow-up plans during the pandemic. These includes postponement of chemotherapy dates for those who were on maintenance phase chemotherapy and dose reduction (one patient). High risk patients (those on intensive phase chemotherapy) were monitored for a longer duration in the hospital while awaiting count recovery prior to discharge, to minimise their risk of getting COVID-19 infection during the myelosuppressive period. Changes to treatment schedules may also be a source of stress to the parents due to the uncertainty whether it will affect their child’s disease outcome. However, due to the small numbers of patients requiring treatment adjustment, we did not perform any statistical analysis to evaluate this further.

Our study was conducted one year after the initial COVID-19 pandemic takes place. New information is released everyday by various sources, but data regarding the impact of COVID-19 infection among paediatric oncology patients remains limited (13). The current study showed less than half (44.9%) of parents knew the effects of COVID-19 infection on children diagnosed with cancer. Study by Darlington et al. during lockdown in England found that 85% of parents or caregivers of childhood cancer patients were worried about the virus and 69.6% felt that the hospital was not a safe place (11). Similarly, a study by Zucchetti et al. in Italy concluded that restrictions to hospital access in Italy during the pandemic increased parents’ psychosocial distress (12). Interestingly, in Netherlands, van Gorp et al. reported fewer caregivers of oncology children were distressed during the early phase of COVID-19 pandemic compared with pre-COVID-19 era (24). The author concluded that instant access to the psycho-oncology department and early reassuring information sharing (i.e., information that suggested that children with cancer seemed relatively unaffected by COVID-19) helped in reducing the parents’ concerns (24).

From the current study, half (54.5%) of the parents were very or extremely stressed when scored using the CSS tool. Low household income was identified as a factor contributing to high stress level among the parents. Previous study before the pandemic by Latiff et al. in one of the study centre revealed that 27.3% parents of children with leukemia had high total parenting stress score (25). However, household income was not analysed in the previous study (25). Two-third of our patients came from the lower socio-economic group who relied heavily on government assistance for hospital bill payments. As hospital social services were considered non-essential during the pandemic, delayed assessment for eligibility for aids have delayed the much-needed assistance to the poor family, possibly contributing to the higher stress level among them. Loss of job by a third of the parents during the pandemic may contribute further to the stress. Hospital social workers can play a bigger role during pandemic by engaging employment agencies as well as non-governmental organizations to identify and offer parents financial assistance and re-employment opportunities in a timely manner.

There are several limitations to our study. Firstly, COVID Stress Scales (CSS) is a new tool with no pre-defined cut-off score to determine low or high stress level. Two authors had used CSS to determine the stress level using different cut-off scores. Marija et al. performed Receiver Operating Characteristic (ROC) curve analysis and determined level above 24.5 as high stress level (specificity of 62.8%; sensitivity of 61.7%) (26). Meanwhile, Taylor et al. used latent class analyses to classify the total CSS score into 5 classes based on mean total score, ranging from low (Class 1) to high (Class 5) stress level (27). Although the CSS is reported to be comparable to another well-established questionnaire, Perceived Stress Scale (PSS), more data is needed to allow better interpretation of the scores obtained (25). Another limitation was the inhomogeneity of patients who participated in this study, with some on different phases of active treatment while others had already completed their treatment. The parents’ stress level would definitely differ between these groups. Furthermore, only one of the parents (majority were mothers, *n* = 135, 80.8%) answered the questionnaire. As both parents may have different stress levels and coping mechanisms, this could affect the CSS results. Lastly, as this study was conducted over a period of 2 years, the parents perception and stress level may have changed at later recruitment timepoint as the COVID-19 cases declined and national policies changed.

## 5 Conclusion

In this study, majority of parents of paediatric oncology patients were aware of COVID-19 infection and its risk to their children. Half of the parents had high stress level during the pandemic. Low household income was found to be associated with high stress level.

## Data availability statement

The raw data supporting the conclusions of this article will be made available by the authors, without undue reservation.

## Ethics statement

Eligible parents were approached and written informed consent was obtained prior to recruitment. Ethics approval was obtained from the Research Ethics Committee, The National University of Malaysia (JEP-2021-028) and The Ministry of Health Medical Research Ethics Committee (MREC) of Malaysia (NMRR-20-3028-56868) prior to the study.

## Author contributions

MA, DL and HA contributed to the conception and design of the study, data analysis, interpretation and writing of the manuscript. MA, SC, KH and BH contributed towards subjects’ recruitment and data collection. All authors contributed to the and approved the submitted version.
