# Supplementary material for: Caring for a child with cancer during COVID-19 pandemic: an assessment of the parents’ perception and stress level
Source: Front Public Health. 2024 Apr 8;12:1223362. doi: 10.3389/fpubh.2024.1223362 (PMC11036860; doi:10.3389/fpubh.2024.1223362)
Supplement: Supplementary file 1 [file Table_1.docx]

**Supplementary data**

Table 1: Covid-19 Stress Scale Scores

|  | Danger | Socio-economic | Xenophobia | Contamination | Traumatic  stress | Compulsive  checking | Total |
| --- | --- | --- | --- | --- | --- | --- | --- |
| Mean score (SD) | 16.77  (4.90) | 10.05  (6.68) | 16.69  (6.17) | 15.62  (5.51) | 6.00  (5.31) | 12.87  (5.09) | 78.01  (25.73) |
| Median score (IQR) | 17.00  (14.00; 20.00) | 11.00  (5.00; 16.00) | 18.00  (13.00; 22.00) | 16.00  (12.00; 19.00) | 5.00  (1.00; 10.00) | 13.00  (10.00; 16.00) | 78.00  (64.00; 95.00) |
| Minimum score | 2.00 | 0.00 | 0.00 | 0.00 | 0.00 | 0.00 | 12.00 |
| Maximum score | 24.00 | 24.00 | 24.00 | 24.00 | 24.00 | 24.00 | 141.00 |

Table 2: Mean score for each item on all CSS component

| **Items (Danger)** | **Mean score (SD)** |
| --- | --- |
| 1. I am worried about catching the virus | 3.16 (0.85) |
| 1. I am worried that I can’t keep my family safe from the virus | 3.04 (0.83) |
| 1. I am worried that our healthcare system won’t be able to protect my loved ones | 2.77 (1.01) |
| 1. I am worried our healthcare system is unable to keep me safe from the virus | 2.70 (1.03) |
| 1. I am worried that basic hygiene (e.g., handwashing) is not enough to keep me safe from the virus | 2.49 (1.08) |
| 1. I am worried that social distancing is not enough to keep me safe from the virus | 2.61 (1.02) |
| **Items (Socio-economic)** |  |
| 1. I am worried about grocery stores running out of food | 1.50 (1.22) |
| 1. I am worried that grocery stores will close down | 1.59 (1.23) |
| 1. I am worried about grocery stores running out of cleaning or disinfectant supplies | 1.79 (1.22) |
| 1. I am worried about grocery stores running out of cold or flu remedies | 1.63 (1.24) |
| 1. I am worried about grocery stores running out of water | 1.60 (1.30) |
| 1. I am worried about pharmacies running out of prescription medicines | 1.93 (1.30) |
| **Items (Xenophobia)** |  |
| 1. I am worried that foreigners are spreading the virus in my country | 2.98 (1.15) |
| 1. If I went to a restaurant that specialized in foreign foods, I’d be worried about catching the virus | 2.53 (1.18) |
| 1. I am worried about coming into contact with foreigners because they might have the virus | 2.81 (1.11) |
| 1. If I met a person from a foreign country, I’d be worried that they might have the virus | 2.93 (1.09) |
| 1. If I was in an elevator with a group of foreigners, I’d be worried that they’re infected with the virus | 2.87 (1.11) |
| 1. I am worried that foreigners are spreading the virus because they’re not as clean as we are | 2.57 (1.18) |
| **Items (Contamination)** |  |
| 1. I am worried that if I touched something in a public space (e.g., handrail, door handle), I would catch the virus | 2.81 (1.00) |
| 1. I am worried that if someone coughed or sneezed near me, I would catch the virus | 3.03 (0.91) |
| 1. I am worried that people around me will infect me with the virus | 2.83 (0.99) |
| 1. I am worried about taking change in cash transactions | 2.33 (1.09) |
| 1. I am worried that I might catch the virus from handling money or using a debit machine | 2.44 (1.07) |
| 1. I am worried that my mail has been contaminated by mail handlers | 2.19 (1.11) |
| **Items (Traumatic stress)** |  |
| 1. I had trouble concentrating because I kept thinking about the virus | 1.56 (1.14) |
| 1. Disturbing mental images about the virus popped into my mind against my will | 1.17 (1.13) |
| 1. I had trouble sleeping because I worried about the virus | 0.92 (1.03) |
| 1. I thought about the virus when I didn’t mean to | 1.12 (1.08) |
| 1. Reminders of the virus caused me to have physical reactions, such as sweating or a pounding heart | 0.79 (0.99) |
| 1. I had bad dreams about the virus | 0.44 (0.84) |
| **Items (Compulsive checking)** |  |
| 1. Searched the Internet for treatments for COVID-19 | 1.91 (1.17) |
| 1. Asked health professionals (e.g., doctors or pharmacists) for advice about COVID-19 | 1.58 (1.15) |
| 1. Checked YouTube videos about COVID-19 | 1.91 (1.11) |
| 1. Checked your own body for signs of infection (e.g., taking your temperature) | 2.46 (1.20) |
| 1. Sought reassurance from friends or family about COVID-19 | 2.22 (0.98) |
| 1. Checked social media posts concerning COVID-19 | 2.80 (1.03) |

Likert scale 0 to 4
