# Supplementary material for: Caring for a child with cancer during COVID-19 pandemic: an assessment of the parents’ perception and stress level
Source: Front Public Health. 2024 Apr 8;12:1223362. doi: 10.3389/fpubh.2024.1223362 (PMC11036860; doi:10.3389/fpubh.2024.1223362)
Supplement: Supplementary file 2 [file Data_Sheet_1.docx]

**QUESTIONNAIRE**

1. Have you heard about the COVID-19 pandemic?

( ) Yes ( ) No

1. Where do you get information about the COVID-19 pandemic?

***(More than one answer is allowable)***

( ) Broadcast media (e.g. radio, television)

( ) Social networks (e.g. facebook, twitter, instagram)

( ) Press conferences or official government statements

( ) Discussions in the family and/or with friends

( ) From doctors / nurses

1. In which aspect is the information you get about the COVID-19 pandemic relevant to you?

***(More than one answer is allowable)***

( ) I know about COVID-19 infections in general

( ) I know about the specific complications of COVID-19 infection on paediatric patients

( ) I know about the specific complications of COVID-19 infection on cancer patients

( ) I know what I need to do to reduce the risk of my child getting COVID-19 infection

( ) I cannot find the information I am looking for

1. Has your child been in contact with a suspected or proven COVID-19 positive patient?

( ) Yes ( ) No

1. If yes, who is this person?

( ) Medical staff

( ) Another patient

( ) Family members/Relatives

( ) Neighbour

( ) Others, please specify _____________

1. Has your child had any COVID-19 swab taken during the pandemic period?

( ) Yes ( ) No

1. If yes, why is it done?

( ) My child had symptoms (fever/cough/etc) but no COVID-19 contact

( ) Suspected due to contact but he/she had no symptom

( ) Screening from the initial hospital before my child was referred to the current treating

hospital

( ) Screening done prior to operation or medical procedure

( ) I had the COVID-19 swab done for my child in private hospital because I am worried

( ) Don’t know (I was not told the indication by the attending doctor)

( ) Other:_________________________________

1. How many times has your child had COVID-19 swab taken?

( ) Once ( ) 2-5 times ( ) More than 5 times

1. Has your child ever been quarantined during this pandemic?

( ) Yes ( ) No

1. If "Yes", why was your child quarantined?

***(More than one answer is allowable)***

( ) My child had symptoms (fever/cough/et cetera)

( ) Contact with COVID-19 patient but no symptoms

( ) When my child was undergoing COVID-19 screening before operation / medical procedure

( ) Don't know (I was not told by the doctor the reason)

( ) Other:______________________________________

1. Do you feel that the COVID-19 pandemic is dangerous to your child who has been diagnosed with cancer?

( ) Dangerous

( ) Moderately or slightly dangerous

( ) Not at all dangerous

( ) Don’t know

1. Are you afraid that your child will catch the COVID-19 infection?

( ) Very afraid

( ) Moderately afraid

( ) A little afraid

( ) Not at all afraid

( ) Don’t know

1. Are you afraid your child might have severe complications if he/she gets the COVID-19 infection?

( ) Very afraid

( ) Moderately afraid

( ) A little afraid

( ) Not at all afraid

( ) Don’t know

1. Have you changed any of your daily habits* pertaining to the care of your child as a result of the COVID-19 pandemic?

( ) Yes

( ) No

*more strict hygiene (hand washing, food preparation), wear masks/enforced child to wear masks

1. If so, what made you change your daily habits pertaining to the care of your child?

***(More than one answer is allowable)***

( ) My choice

( ) Imposed by my family

( ) Imposed by the institutions

( ) Based on what others do

( ) Don’t know

1. Did you see any changes in the health personnel’s practice during coronavirus pandemic when handling your child?

( ) Yes

( ) No

1. If your answer is "Yes" in question number 24, can you elaborate further?

________________________________________________________________________

________________________________________________________________________

1. Do you think this pandemic has any positive consequences on your child’s care?

***(More than one answer is allowable)***

( ) Medical personnel wash their hand more often before touching my child

( ) Medical personnel wearing a mask almost all the time

( ) I see medical personnel and everyone practising social distancing in hospital

( ) It’s all the same to me

( ) Don’t think so/don’t know

( ) Others, please state ____________________________________________________

1. Would you like to have more information about the issue?

( ) Yes ( ) No

1. How does this pandemic affect your financial status?

( ) Major impact (Please specify on the next question)

( ) Slight impact (I can still afford my child’s cost of treatment and my family’s costof daily living if I am careful with my money)

( ) No impact at all

1. If this pandemic give you a major impact on your financial status, please specify:

***(Can choose more than 1 answer)***

( ) I lost my job and income

( ) My monthly salary is cut

( ) I had to use almost all of my saving (KWSP)

( ) I had to borrow money from others/request assistance from hospital’s Medical Social Work Services (JKSP) to support my child’s cost of treatment.

( )I had to borrow money from others to support my family’s cost of daily living.

1. Did you face any difficulties bringing your child to the hospital during the pandemic?

( ) Yes ( ) No

1. If "Yes", what difficulties did you encounter?

***(Can choose more than 1 answer)***

( ) Public transport services (bus, KTM) are not available during MCO

( ) Public transport services are available but I do not feel safe using them

( ) Taxi / GRAB services are too expansive for me

( ) It’s all the same to me

( ) I am late or cannot bring my child to hospital because of police road block

( ) I am not allowed to travel across state to bring my child to hospital

( ) Other:__________________________________________________

***For patients who are still on treatment. Please answer question 32 until 37.***

1. Does your doctor make any modification for your child’s cancer treatment during this pandemic?

( ) Yes

( ) No

( ) Don’t know (I was not aware of this)

1. If "Yes", what modification was done to your child’s cancer treatment? (eg. procedure/ appointment postponed to a later date, chemotherapy dose reduced, treatment withheld temporarily, et cetera)

________________________________________________________________________

________________________________________________________________________

1. Do you think the modification is required for your child’s treatment during the pandemic?

( ) Yes ( ) No

***For patients who have completed treatment and are on follow-up in clinic. Please answer question number 38 until 44.***

1. If your child is on follow up, does the doctor made any changes to your child’s follow up?

( ) Yes

( ) No

( ) Don’t know (I was not aware of this)

1. If yes, what change was made to your child’s follow up?

***(more than one answer is allowable)***

( ) Ultrasound/CT scan/MRI delayed

( ) Less frequent blood taking

( ) Longer follow up interval

( ) I need to go to other hospital

( ) Other, please state ______________________

1. Do you think the modification is required for your child’s treatment during the pandemic?

( ) Yes ( ) No

**COVID Stress Scale (CSS)**

**ARAHAN:** Berikut ini adalah pernyataan tentang pelbagai jenis kebimbangan yang mungkin anda alami dalam tujuh hari lepas. Dalam pernyataan berikut, kami merujuk COVID-19 sebagai "virus".

*The following asks about various kinds of worries that you might have experienced over the past seven days. In the following statements, we refer to COVID-19 as "the virus".*

| 0 = Tidak sama sekali | *0 = Not at all* |
| --- | --- |
| 1 = Sedikit | *1 = Slightly* |
| 2 = Sederhana | *2 = Moderately* |
| 3 = Sangat | *3 = Very* |
| 4 = Ekstrem | *4 = Extremely* |

| **Item** | | **0** | | **1** | | **2** | | **3** | | **4** |
| --- | --- | --- | --- | --- | --- | --- | --- | --- | --- | --- |
| 1. Saya bimbang tentang dijangkiti virus ini   *I am worried about catching the virus* | |  | |  | |  | |  | |  |
| 1. Saya bimbang bahawa saya tidak dapat menjaga keselamatan keluarga daripada virus ini   *I am worried that I can’t keep my family safe from the virus* | |  | |  | |  | |  | |  |
| 1. Saya bimbang bahawa sistem penjagaan kesihatan kita tidak dapat melindungi orang yang saya sayangi   *I am worried that our healthcare system won’t be able to protect my loved ones* | |  | |  | |  | |  | |  |
| 1. Saya bimbang sistem penjagaan kesihatan kita tidak dapat melindungi saya daripada virus ini   *I am worried our healthcare system is unable to keep me safe from the virus* | |  | |  | |  | |  | |  |
| 1. Saya bimbang bahawa penjagaan asas kebersihan (cth: mencuci tangan) tidak mencukupi untuk melindungi saya daripada virus ini   *I am worried that basic hygiene (e.g., handwashing) is not enough to keep me safe from the virus* | |  | |  | |  | |  | |  |
| **Item** | | **0** | | **1** | | **2** | | **3** | | **4** |
| 1. Saya bimbang bahawa penjarakan sosial tidak mencukupi untuk melindungi saya daripada virus ini   *I am worried that social distancing is not enough to keep me safe from the virus* | |  | |  | |  | |  | |  |
| 1. Saya bimbang tentang kedai runcit akan kehabisan makanan   *I am worried about grocery stores running out of food* | |  | |  | |  | |  | |  |
| 1. Saya bimbang bahawa kedai runcit akan tutup   *I am worried that grocery stores will close down* | |  | |  | |  | |  | |  |
| 1. Saya bimbang tentang kedai runcit akan kehabisan bekalan bahan untuk pembersihan atau penyahjangkitan   *I am worried about grocery stores running out of cleaning or disinfectant supplies* | |  | |  | |  | |  | |  |
| 1. Saya bimbang tentang kedai runcit akan kehabisan ubat demam atau selsema   *I am worried about grocery stores running out of cold or flu remedies* | |  | |  | |  | |  | |  |
| 1. Saya bimbang tentang kedai runcit akan kehabisan bekalan air   *I am worried about grocery stores running out of water* | |  | |  | |  | |  | |  |
| 1. Saya bimbang tentang farmasi kehabisan ubat yang dipreskripsi   *I am worried about pharmacies running out of prescription medicines* | |  | |  | |  | |  | |  |
| 1. Saya bimbang bahawa warga asing menyebarkan virus di negara saya   *I am worried that foreigners are spreading the virus in my country* | |  | |  | |  | |  | |  |
| 1. Jika saya pergi ke restoran yang khusus dalam penyediaan makanan negara lain, saya akan bimbang tentang dijangkiti virus ini   *If I went to a restaurant that specialized in foreign foods, I’d be worried about catching the virus* | |  | |  | |  | |  | |  |
| 1. Saya bimbang tentang berhubung dengan warga asing kerana mereka mungkin mempunyai virus ini   *I am worried about coming into contact with foreigners because they might have the virus* | |  | |  | |  | |  | |  |
| **Item** | | **0** | | **1** | | **2** | | **3** | | **4** |
| 1. Jika saya bertemu seseorang daripada luar negara, saya akan bimbang bahawa mereka mungkin mempunyai virus ini   *If I met a person from a foreign country, I’d be worried that they might have the virus* | |  | |  | |  | |  | |  |
| 1. Jika saya berada di dalam lif dengan sekumpulan warga asing, saya akan bimbang bahawa mereka telah dijangkiti virus ini   *If I was in an elevator with a group of foreigners, I’d be worried that they’re infected with the virus* | |  | |  | |  | |  | |  |
| 1. Saya bimbang bahawa warga asing menyebarkan virus ini kerana mereka tidak sebersih kita   *I am worried that foreigners are spreading the virus because they’re not as clean as we are* | |  | |  | |  | |  | |  |
| 1. Saya bimbang bahawa jika saya menyentuh sesuatu di tempat awam (cth: pemegang tangan, pemegang pintu), saya akan dijangkiti virus ini   *I am worried that if I touched something in a public space (e.g., handrail, door handle), I would catch the virus* | |  | |  | |  | |  | |  |
| 1. Saya bimbang bahawa jika seseorang yang berhampiran dengan saya batuk atau bersin, saya akan dijangkiti virus ini   *I am worried that if someone coughed or sneezed near me, I would catch the virus* | |  | |  | |  | |  | |  |
| 1. Saya bimbang bahawa orang disekeliling saya akan menjangkiti saya dengan virus ini   *I am worried that people around me will infect me with the virus* | |  | |  | |  | |  | |  |
| 1. Saya bimbang tentang mengambil baki ketika urusan transaksi tunai   *I am worried about taking change in cash transactions* | |  | |  | |  | |  | |  |
| 1. Saya bimbang bahawa saya mungkin dijangkiti virus ini daripada penggendalian wang atau menggunakan mesin debit   *I am worried that I might catch the virus from handling money or using a debit machine* | |  | |  | |  | |  | |  |
| 1. Saya bimbang bahawa surat saya telah dicemari oleh pengendali surat   *I am worried that my mail has been contaminated by mail handlers* | |  | |  | |  | |  | |  |
| Sila baca setiap kenyataan di bawah dan nyatakan kekerapan anda mengalami masalah tersebut sepanjang tempoh tujuh hari yang lalu.  *Please read each statement and indicate how frequently you have experienced each problem during the past seven days.*   \| 0=Tidak pernah \| *0 = Never* \| \| --- \| --- \| \| 1=Jarang \| *1 = Rarely* \| \| 2=Kadangkala \| *2 = Sometimes* \| \| 3=Selalu \| *3 = Often* \| \| 4=Hampir selalu \| *4 = Almost always* \| | | | | | | | | | | |
| **Item** | **0** | | **1** | | **2** | | **3** | | **4** | |
| 1. Saya mempunyai masalah menumpukan perhatian kerana saya sentiasa berfikir tentang virus ini   *I had trouble concentrating because I kept thinking about the virus* |  | |  | |  | |  | |  | |
| 1. Imej gangguan mental yang berkaitan dengan virus ini muncul dalam fikiran saya berlawanan dengan kehendak saya   Disturbing mental images about the virus popped into my mind against my will |  | |  | |  | |  | |  | |
| 1. Saya mengalami masalah tidur kerana saya bimbang tentang virus ini   *I had trouble sleeping because I worried about the virus* |  | |  | |  | |  | |  | |
| 1. Saya berfikir tentang virus ini bila saya tidak bermaksud untuk memikirkannya   *I thought about the virus when I didn’t mean to* |  | |  | |  | |  | |  | |
| 1. Peringatan berkaitan virus ini menyebabkan saya mengalami reaksi fizikal, seperti berpeluh atau jantung berdebar-debar   *Reminders of the virus caused me to have physical reactions, such as sweating or a pounding heart* |  | |  | |  | |  | |  | |
| 1. Saya mengalami mimpi buruk tentang virus ini   *I had bad dreams about the virus* |  | |  | |  | |  | |  | |
| Item berikut adalah berkaitan soalan tentang pemeriksaan tingkah laku. Sepanjang tujuh hari yang lalu, berapa banyak anda telah melakukan yang berikut kerana keperihatinan tentang COVID-19?  *The following items ask about checking behaviours. During the past seven days, how much have you done the following because of concerns about COVID-19?*   \| 0=Tidak pernah \| *0 = Never* \| \| --- \| --- \| \| 1=Jarang \| *1 = Rarely* \| \| 2=Kadangkala \| *2 = Sometimes* \| \| 3=Selalu \| *3 = Often* \| \| 4=Hampir selalu \| *4 = Almost always* \| | | | | | | | | | | |
| **Item** | **0** | | **1** | | **2** | | **3** | | **4** | |
| 1. Melayari internet untuk rawatan COVID-19   *Searched the Internet for treatments for COVID-19* |  | |  | |  | |  | |  | |
| 1. Meminta nasihat pakar kesihatan (cth: doktor atau ahli farmasi) tentang COVID-19   *Asked health professionals (e.g., doctors or pharmacists) for advice about COVID-19* |  | |  | |  | |  | |  | |
| 1. Menyemak video YouTube tentang COVID-19   *Checked YouTube videos about COVID-19* |  | |  | |  | |  | |  | |
| 1. Memeriksa badan sendiri untuk gejala jangkitan (cth: mengambil suhu badan sendiri)   *Checked your own body for signs of infection (e.g., taking your temperature)* |  | |  | |  | |  | |  | |
| 1. Mendapatkan kepastian daripada rakan atau keluarga tentang COVID-19   *Sought reassurance from friends or family about COVID-19* |  | |  | |  | |  | |  | |
| 1. Menyemak siaran media sosial berkaitan COVID-19   *Checked social media posts concerning COVID-19* |  | |  | |  | |  | |  | |
